# Quantifying the contribution of changes in healthcare expenditures and smoking to the reversal of the trend in life expectancy in the Netherlands

**DOI:** 10.1186/s12889-015-2357-2

**Published:** 2015-10-06

**Authors:** Frederik Peters, Wilma J. Nusselder, Nadine Reibling, Christian Wegner-Siegmundt, Johan P. Mackenbach

**Affiliations:** Department of Public Health, Erasmus MC, University Medical Center Rotterdam, PO Box 2040, 3000 CA Rotterdam, The Netherlands; Department of Health Policy and Management, Harvard School of Public Health, Boston, USA; Wittgenstein Centre for Demography and Global Human Capital (IIASA, VIS/ÖAW, WU), Vienna Institute of Demography/Austrian Academy of Sciences, Vienna, Austria

**Keywords:** Healthcare systems, Life expectancy, Smoking

## Abstract

**Background:**

Since 2001 the Netherlands has shown a sharp upturn in life expectancy (LE) after a longer period of slower improvement. This study assessed whether changes in healthcare expenditure (HCE) explain this reversal in trends in LE. As an alternative explanation, the impact of changes in smoking behavior was also evaluated.

**Methods:**

To quantify the contribution of changes in HCE to changes in LE, we estimated a health-production function using a dynamic panel regression approach with data on 19 OECD countries (1980–2009), accounting for temporal and spatial correlation. Smoking-attributable mortality was estimated using the indirect Peto-Lopez method.

**Results:**

As compared to 1990–1999, during 2000–2009 LE in the Netherlands increased by 1.8 years in females and by 1.5 years in males. Whereas changes in the impact of smoking between the two periods made almost no contribution to the acceleration of the increase in LE, changes in the trend of HCE added 0.9 years to the LE increase between 2000 and 2009. The exceptional reversal in the trend of LE and HCE was not found among the other OECD countries.

**Conclusion:**

This study suggests that changes in Dutch HCE, and not in smoking, made an important contribution to the reversal of the trend in LE; these findings support the view that investments in healthcare are increasingly important for further progress in life expectancy.

**Electronic supplementary material:**

The online version of this article (doi:10.1186/s12889-015-2357-2) contains supplementary material, which is available to authorized users.

## Background

Since the 19th century life expectancy at birth has increased dramatically in Western high-income countries [1]. During the second part of the 20th century the rate of increase in most of these countries was very similar, with no disruptions or signs of slowing down [2, 3]. This remarkable finding led to the belief that progress in survival is a universal feature largely independent of country-specific aspects, such as the set-up of the health system or differences in health-specific behavior [2]. However, this hypothesis was challenged by some particularly successful economies witnessing longer periods with a slower increase, or even stagnation, in life expectancy [4–6].

An example for such a country is presented by the Netherlands, where mortality declined more slowly than in other countries during the 1980s and 1990s, particularly for women [7]. However, in the year 2002 a sudden and strong increase in life expectancy started and has continued until today [8]. One hypothesis for this reversal in trend is that additional investments in the health sector led to improvements in survival, particularly at older ages [8]. On the other hand, an exceptionally high impact of damage caused by smoking has frequently been mentioned as a competing explanation, particularly relevant during the stagnation period of Dutch life expectancy improvements [4, 9]. Despite considerable research, no convincing evidence is available on the factors behind the stagnation period and the subsequent period of resumption of the improvement in Dutch life expectancy [10].

Therefore, this study is the first to quantify the impact of healthcare expenditure on the change from a slower increase to a rapid improvement in Dutch life expectancy, while also assessing the contribution of smoking as an alternative explanation. Additionally, we evaluated whether the internationally deviating trends in Dutch life expectancy corresponded to internationally deviating trends in healthcare expenditures or smoking.

For this purpose, we compared the results from the Netherlands with a group of 18 comparable countries of the Organization for Economic Co-operation and Development (OECD) based on data covering life expectancy, lung cancer mortality, healthcare expenditures and gross domestic product for the years 1980–2009. To estimate the impact that changes in healthcare expenditures had on life expectancy we used a panel data analysis accounting for unobserved factors, cross-country variation, and dynamic effects. The impact of smoking was estimated by the indirect Peto-Lopez method.

## Methods

### Data collection

Countries included in the analysis were all members of the OECD since (at least) 1980, because the annual provision of country-specific data is legally linked to this membership status. Excluded were Luxembourg because it provides data on healthcare expenditures from 1999 onwards only, and the USA due to its fundamentally different healthcare system [11]. This leaves the following 19 countries for analysis: Australia, Austria, Belgium, Canada, Denmark, Finland, France, Iceland, Ireland, Italy, Japan, the Netherlands, Norway, New Zealand, Portugal, Spain, Sweden, Switzerland and the United Kingdom.

Information on mortality rates for the ages 0 to 100 in 5-year age groups were obtained from the Human Mortality Database [12]. Sex-specific lung cancer death rates in 5-year age groups for ages 35 to 89 years were taken from the WHO mortality database to obtain smoking-attributable fractions [13]. To model the influence of healthcare expenditures on life expectancy at birth, we collected data on healthcare expenditures and gross-domestic product (GDP) for the years 1980–2009 from the 2014 OECD Health Data collection [14].

### Statistical analysis

The analysis quantified the contribution of changes in smoking and healthcare expenditures to changes of life expectancy at birth, between 1990 and 2009, using two different techniques.

The impact of smoking on mortality was estimated using the validated indirect Peto-Lopez method, which utilizes lung cancer death rates as an indicator for the cumulative damage of smoking to all other causes of death on the basis of relative risks obtained from a large cohort study [15]. This resulted in country-specific annual smoking-attributable fractions in 5-year age groups from age 35 to 85 years and an open-ended category 85+. These fractions were used to remove smoking-related mortality from the observed mortality rates, that were also tabulated in annual 5-year age groups to compute smoking-free life expectancy applying life table methods. The smoking-attributable fraction at age 85+ years was applied only to mortality between age 85–90 years because, at older ages, the impact of smoking is very small and cause-of-death statistics are less trustworthy [15]. For a few calendar years for which lung cancer deaths were missing, we interpolated smoking-attributable fractions using local polynomial regression fitting.

Because competing strategies to estimate the impact of healthcare expenditures on life expectancy have been proposed in the literature [16–18], we performed a separate analysis beforehand comparing different model approaches (presented in detail in the Additional file 1). In brief, we estimated a health-production function relating monetary inputs in healthcare to gains in life expectancy building on recent developments in the analysis of relationships in panel data. Specifically, we modelled a dynamic response of life expectancy to changes in healthcare spending and allowed for heterogeneity in this relation between countries [18]. Thereby, the model tests whether there is an immediate and/or delayed effect of changes in healthcare spending on changes in life expectancy by merely assuming that the size of the delayed effect declines geometrically over time. Moreover, we included spatially correlated common factors in the production function accounting for the fact that developments in the countries do not occur independently of each other [17]. Our theoretically preferred model was compared to alternative specifications on the basis of model fit and panel residual diagnostics. In our main analysis, we multiplied the parameters of this preferred model with the changes in country-specific healthcare expenditures to quantify their impact on life expectancy between 1980 and 2009.

Finally, we compared the gain in life expectancy in 1990–1999 and in 2000–2009 with the gains attributed to changes in smoking and with gains due to increases in healthcare expenditures for the Netherlands and the average of the other 18 countries. Thereby, life expectancy at birth was computed using life table analysis [19]. Although the trend break in Dutch life expectancy occurred around 2002 and not in 2000, for the sake of more robust results we decided to compare the most recent decade to the preceding one so that the analysis covered 10 full calendar years respectively. We computed 95 % confidence intervals (CIs) around the estimates of the impact of healthcare expenditures since their contribution is more uncertain than the contribution of smoking. This was performed by means of simulation (10000 runs) using the variance-covariance matrix of the panel regression results. We used STATA 13 and GNU R 3.1.1 for the calculations.

## Results

### Descriptive trends

**Fig. 1:**
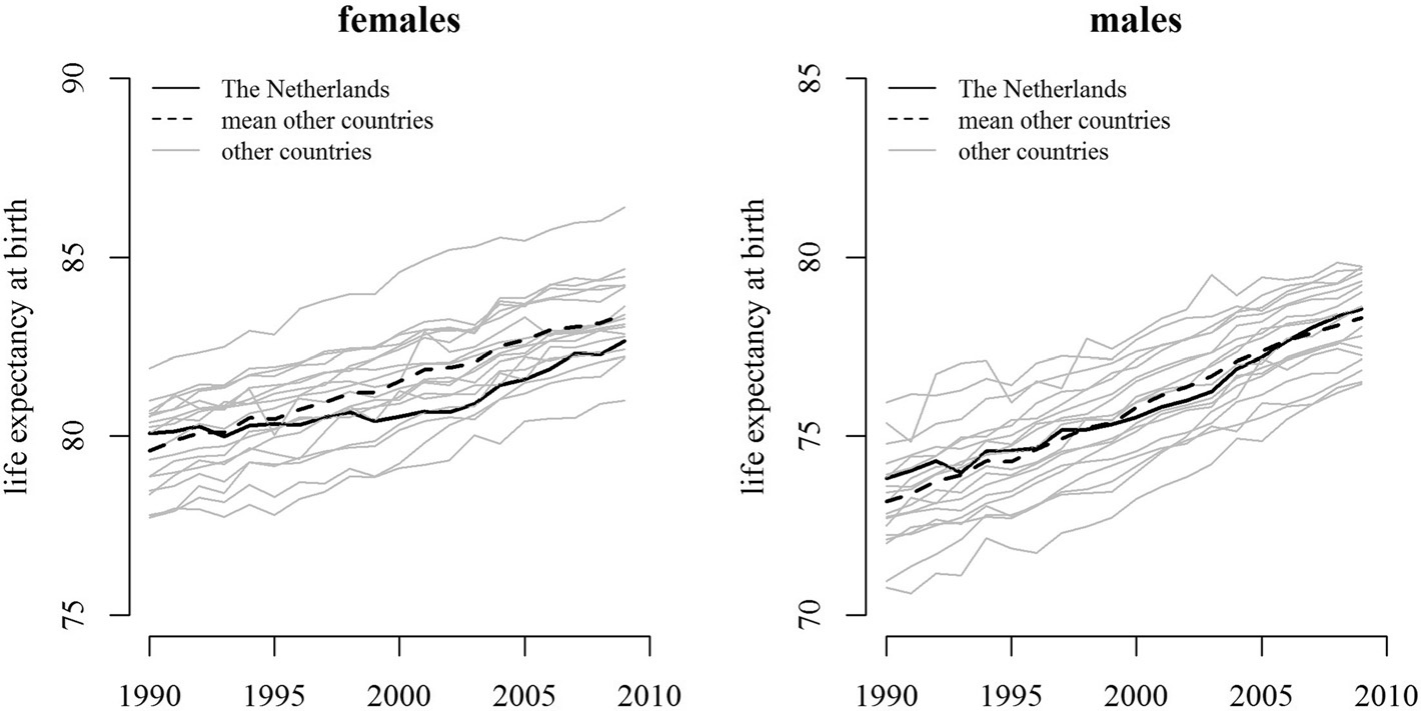
Trends in female and male life expectancy at birth in the Netherlands and in 18 other OECD countries between 1990 and 2009

Comparing the Netherlands to the average of the other 18 OECD countries shows that Dutch life expectancy increases at a slower rate up until about 2002 and faster thereafter, which was more pronounced for females (Fig. 1).

**Fig. 2:**
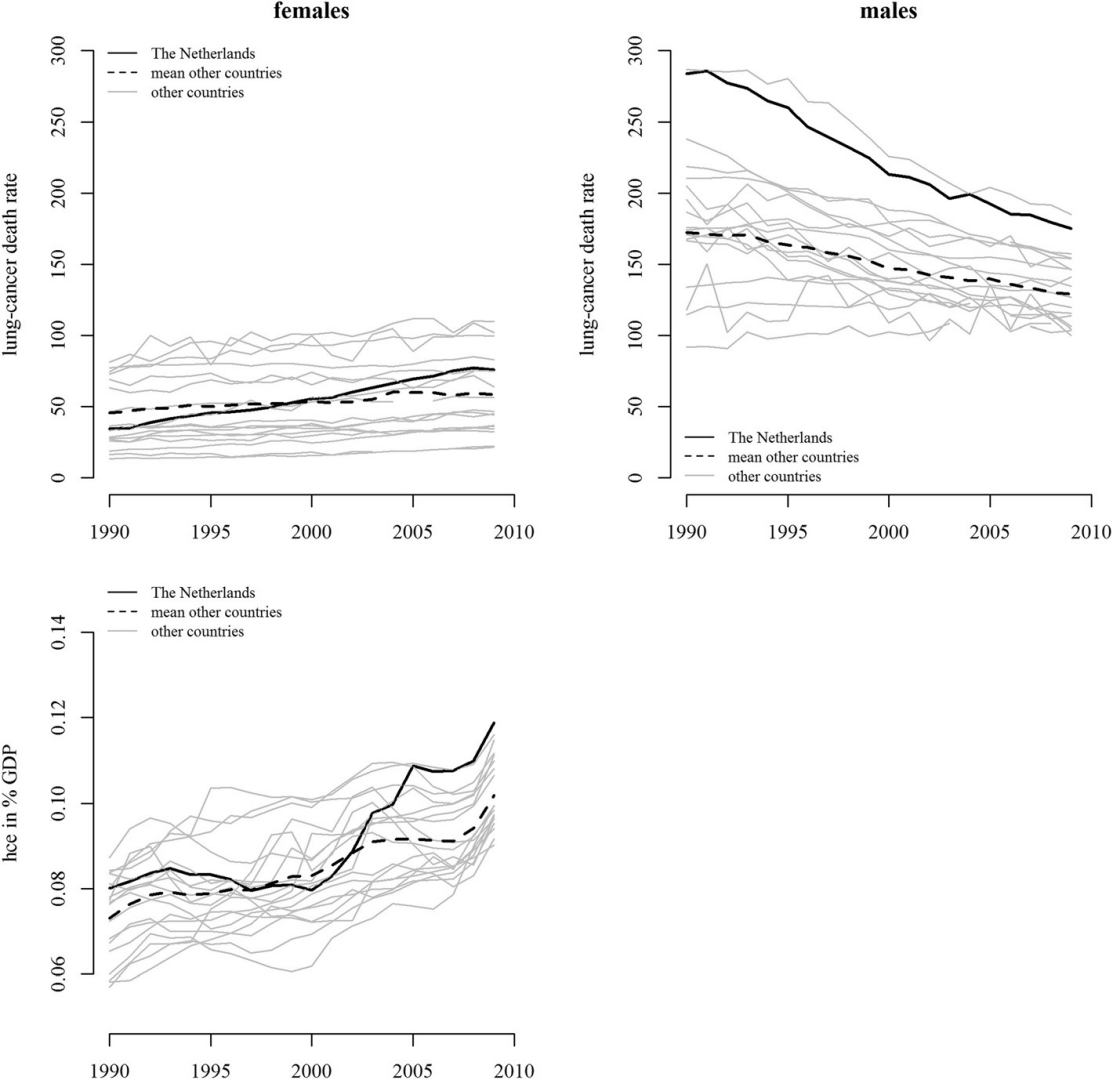
Change in age-standardized lung cancer death rate between age 35 and 89 per 100,000 persons in females and males (upper panel) and healthcare expenditures measured in percentage of gross-domestic product (GDP) (lower panel) in the Netherlands (solid line) and the mean of 18 other OECD countries (dashed line) between 1990 and 2009. Note: Lung-cancer death rates were age-standardized based on the 2013 European Standard Population for illustrative purposes only

Trends in the age-standardized lung cancer death rate, which served as input for the estimation of smoking-associated mortality, reveal large gender differences (Fig. 2). The exceptionally high lung cancer death rate in Dutch males in 1990 decreased rapidly over time, while Dutch females exhibited increasing rates during the entire study period.

The pattern of trends in healthcare expenditures partly resembled the pattern of trends in life expectancy (Fig. 2): expressed as a proportion of the GDP, healthcare expenditures in the Netherlands stagnated up until 2001 and rose thereafter, whereas in the other countries there was a continuous increase over time.

### Effect of changes in healthcare expenditures on life expectancy

A dynamic relationship between healthcare expenditures and life expectancy was confirmed in our sample of 19 countries in 1980–2009 (see Additional file 1): a 10 % increase in healthcare expenditures translates into an increase of life expectancy of 0.36 % on the long term (95 % CI: 0.26-0.58). Given a level of life expectancy of 80 years this would amount to an increase of 0.29 years or 3.5 months.

### Impact of changes in healthcare expenditures and smoking on changes in life expectancy

**Fig. 3:**
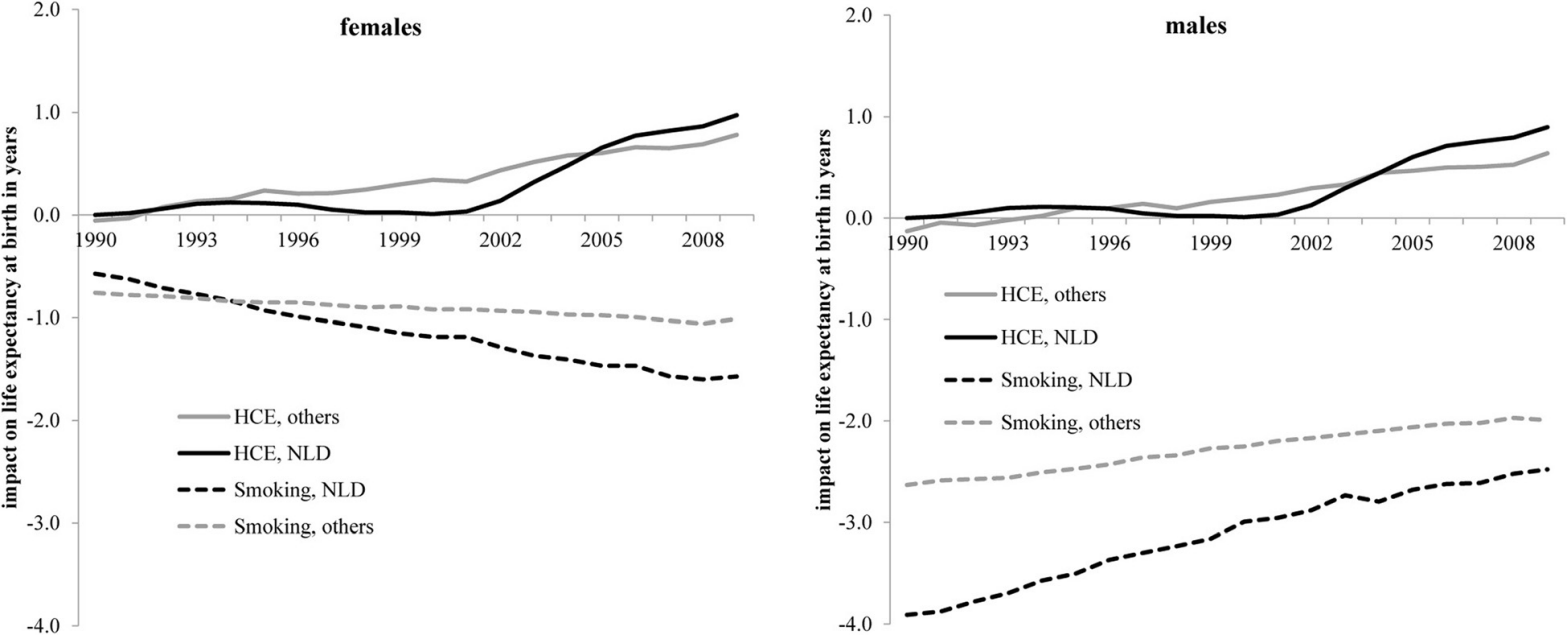
Estimated impact of changes in smoking and healthcare expenditures on life expectancy at birth in females (left panel) and males (right panel) in the Netherlands and the mean of 18 other OECD countries between 1990 and 2009

The contribution of changes in healthcare expenditures to changes in life expectancy is shown in Fig. 3. While Dutch females and males gained about one year of life between 1990 and 2009, this gain was more modest for the other OECD countries on average. The impact of healthcare spending in the Netherlands remained stable until about 2000 followed by a rapid increase thereafter. On average the other countries show a continuous linear increase.

The impact of changes in smoking on changes in life expectancy between 1990 and 2009 occurred in a linear manner for both the Netherlands and the mean of the other countries (Fig. 3). Dutch women suffered above-average losses of years of life due to more damage from smoking while Dutch men gained above-average years of life due to less damage from smoking.

Table 1 summarizes the impact of changes in smoking and healthcare expenditures. Compared to the period 1990–1999, in the period 2000–2009 the change in Dutch life expectancy accelerated by 1.8 years in females and by 1.5 years in males. Changes in healthcare expenditures contributed 0.9 years to this trend, whereas changes in smoking had practically no impact. Taking into account the uncertainty around the estimates, a conservative estimate would ascribe at least 20 % of the acceleration in the improvement of Dutch life expectancy (0.29 years in females and 0.27 years in males) to larger changes in healthcare spending.

**Table 1:** Decennial change in life expectancy (LE) at birth and contribution of smoking and healthcare expenditures (HCE) in the Netherlands and the mean of 18 other OECD countries, 1990–1999 and 2000–2009

	Period	Observed change in LE	Difference between 1990–1999 and 2000–2009	Change in LE due to changes in smoking	Difference between 1990–1999 and 2000–2009	Change in LE due to changes in HCE	95 % CI	Difference between 1990–1999 and 2000–2009	95 % CI
Females		The Netherlands							
	1990–99	0.3		−0.6		0.0	(0.01 to 0.05)		
	2000–09	2.1	1.8	−0.4	0.2	1.0	(0.30 to 1.62)	0.9	(0.29 to 1.57)
		Mean of the other countries							
	1990–99	1.7		−0.1		0.3	(0.09 to 0.52)		
	2000–09	1.9	0.2	−0.1	0.0	0.5	(0.14 to 0.77)	0.2	(0.05 to 0.25)
Males		The Netherlands							
	1990–99	1.5		0.7		0.0	(0.01 to 0.04)		
	2000-09	3.0	1.5	0.5	−0.2	0.9	(0.27 to 1.49)	0.9	(0.27 to 1.45)
		Mean of the other countries							
	1990–99	2.2		0.4		0.3	(0.09 to 0.47)		
	2000–09	2.5	0.3	0.3	−0.1	0.4	(0.13 to 0.70)	0.1	(0.00 to 0.20)

On average there were no large differences in the change of life expectancy or in the change of the impact of smoking and healthcare expenditures in the group of the other countries. Interestingly, the slightly larger change in life expectancy during 2000–2009 as compared to 1990–1999 can almost fully be attributed to a slightly larger change of healthcare expenditures.

### Sensitivity analysis

To assess the robustness of our results, we used an alternative indicator for healthcare expenditures (per capita healthcare expenditures expressed in US$) and estimated smoking-attributable fractions with a different regression-based approach, as suggested by Preston and colleagues [20]. This did not substantially change the main findings of our analysis (see Additional file 1).

Also, we portioned the data into two different segments directly covering the years before (1994–2001) and after (2002–2009) the trend reversal in life expectancy occurred. Although this alternative analysis covered fewer years of observation (16 instead of 20 years in total), the estimates were virtually the same (see Additional file 1).

## Discussion

This study is the first quantitative assessment of the contribution of healthcare expenditures to the recent trend reversal of Dutch life expectancy, also accounting for the contribution of smoking. Our results suggest that changes in healthcare expenditures contributed largely to the trend reversal, with hardly any contribution being made by changes in smoking.

Moreover, the exceptionally large impact of changes in healthcare expenditures on life expectancy were indeed a unique feature of the Netherlands and not present to the same extent in our comparison group of 18 OECD countries.

### Evaluation of data

Since we included only high-income countries with well-established systems of national statistics that were members of the OECD for at least as long as the study period, we are confident about the comparability and quality of the data in general. Missing information that often casts doubt on international comparison was not an issue, since our sample had almost complete information on the study variables [18]. Data on all-cause mortality and population exposure, as well as healthcare expenditures and GDP, were obtained from harmonized databases. Nevertheless, some caveats apply for certain aspects of the data. The implementation of the OECD system of health accounts framework underlying the data on healthcare expenditures varied among countries and over time limiting the comparability. Thereby, differences in the definition of costs for long-term care were identified as most important issue affecting the share of HCE to GDP up to 1 percentage points [21]. To cope with such inconsistencies, we used different statistical models and sample compositions, suggesting that our results are generally robust against these inconsistencies (see Additional file 1). Information on lung cancer counts is (as is the case with all cause-of-death specific data) subject to uncertainty due to variations in national coding practices and changes in coding behavior over time [22]. However, these issues are of minor relevance for lung cancer data because detection is relatively clear and coding schemes have been established for a long time without drastic changes [23].

### Evaluation of methods

The most important part of our study is the quantification of the effect of changes in healthcare expenditures on changes in life expectancy. Although there is a longer tradition of assessing this relationship in empirical research, there is no consensus on the appropriate strategy to estimate it [16]. We believe this is largely due to the absence of appropriate tools to analyze panel data, that became available only a few years ago [18, 24]. A main insight of this new literature is that the relation between healthcare expenditures and life expectancy can be estimated more reliably for a group of countries than for an individual country alone [25]. However, this comes at the cost of providing only “insights regarding the central tendency of the panel” so that we had to apply the same parameter for every country in the main part of our analysis to compute the contribution of healthcare expenditures on life expectancy [26]. Nevertheless, this assumption seems plausible for the countries in our sample, as they share similar political and economic structures, and all provide almost full public coverage of basic healthcare services [27]. If there were substantial differences in the efficiency of the production of health between the Netherlands and the other 18 countries, this would be likely within the relatively large confidence intervals around our main results representing the large uncertainty about the effect of changes in healthcare expenditures.

In our analysis, we assumed that variations in healthcare spending causally explain variations in life expectancy ruling out the opposite direction, i.e., that improvements in life expectancy cause additional costs in the health sector. A study explicitly testing the influences of such reverse causality found that improvements in most health outcomes did not led to higher healthcare costs [28]. Furthermore, it is reported that with postponing death also costs are postponed, deeming increases in life expectancy to be less relevant for growing healthcare expenditures [29]. An exception is spending on long-term care that is to a large extent driven by population ageing [30]. Evaluating the influence of long-term care on healthcare expenditures on our estimates is hardly possible since this aspect of the OECD data is particularly imprecisely measured, as explained above.

Regarding the effect of smoking, we applied a well-established tool that has proven reliable and informative in numerous applications [31, 32]. Although the indirect modeling of the damage from smoking on other causes-of-death partly relies on a set of assumptions, different approaches with different assumption arrived at very similar estimates [20, 33]. Furthermore, the estimated smoking-attributable fractions plausibly describe the variation of the timing in the epidemiological transitions between countries and sexes [32, 34].

An important assumption of our study is that the contribution of healthcare expenditures and smoking on trends in life expectancy could be quantified independently of each other. This might be a simplistic view, given that a well-funded healthcare system certainly mitigates the consequences of smoking, particularly for smoking-induced cardiovascular diseases for which effective treatments exist. However, due to the lack of evidence on that topic, it is difficult to speculate on the impact of our assumption on the study results.

### Comparison with other studies

The results of this study underpin the hypothesis that changes in healthcare expenditures were the main driver of the trend reversal of life expectancy in the Netherlands [8]. While previous analyses only demonstrated the presence of a common trend break for these two variables at around 2002, our study shows that: 1) changes in healthcare spending were generally positively associated with changes in life expectancy within high-income countries, 2) the size of the impact of healthcare could plausibly explain the acceleration of the Dutch life expectancy increase, 3) the exceptionally large changes in healthcare spending and the trend reversal in life expectancy at around 2002 was a particular feature of the Netherlands, and 4) that the major alternative explanation for the trend reversal, i.e., changes in smoking, could be ruled out. These four aspects explaining the Dutch trend reversal counter arguments stating: *“that there is no observable relationship with changes healthcare funding whatsoever*” [10].

The importance of the contribution of healthcare expenditures to the trend reversal in Dutch life expectancy is in line with case studies of other countries with rapid trend reversals in life expectancy. The natural experiment of the separation and subsequent unification of Germany demonstrated that improvements of the healthcare infrastructure could affect life expectancy immediately and with a large impact [35]. In Denmark, a huge investment program to reduce cardiovascular mortality was held partially responsible for the upturn in Danish life expectancy after a longer period of stagnation [36]. The same was noted for the case of Ireland, where particularly cardiovascular mortality suddenly declined after access to primary care and pharmaceuticals was facilitated in 2001 [37]. The results of our main analysis confirm the findings for Denmark and Ireland that increases in healthcare expenditures in these countries contributed to the accelerations of the increase in life expectancy (see Additional file 1).

Nevertheless, the example of Japan shows that a large increase in life expectancy can be achieved without a large increase in healthcare spending, while the example of the USA shows that substantial investments in healthcare spending do not necessarily lead to large improvements in life expectancy [38, 39]. This highlights the relevance of contextual factors, such as a universal coverage of healthcare services or more general cultural aspects. Clearly, investments in healthcare systems have a larger impact on life expectancy in countries where costly but also effective medical treatments were held back.

Our results attributed a considerable part of the increase in Dutch life expectancy to changes in Dutch healthcare expenditures, i.e., about 47 % for Dutch women and about 30 % for Dutch men in 2000–2009. Studies that combined knowledge on the effect of new medical treatments with cause-specific mortality data and disease prevalence, estimated that innovations in healthcare have contributed to at least 50 % of the gains in life expectancy during recent decades, which is in line with our estimates [40].

### Explanations of findings

A major way in which variations in Dutch healthcare expenditures could have affected mortality, is the budgeting of hospital care. During the early 1980s, at the time the improvements in Dutch life expectancy started to slow down, policymakers introduced fixed hospital budgets resulting in a considerable reduction in the admission of new patients, of employed personnel and even in the closure of some hospitals [41, 42]. To cope with the budgeting of their resources hospitals invested less in new medical technologies and limited the volume of treatment for their patients [42]. Together with the legalization of euthanasia in 1985 and an increasing incidence of end-of-life decisions (e.g., the withdrawal of artificial nutrition), this reflected a general attitude towards a less aggressive treatment of older and terminally-ill patients [7]. Consequently, the dramatic improvements in survival due to innovations in the treatment of cardiovascular diseases observed in many Western countries particularly at older ages, where probably not fully realized in the Netherlands where mortality rates even increased for some age groups [3, 4].

At the end of the 1990s, complaints about excessive waiting times for elective surgeries piled up, so that policymakers abolished the fixed hospital budgets at the end of 2000 and replaced them with activity-based funding [43]. As a result hospital admissions, treatments, and pharmaceutical prescriptions increased rapidly, particularly among the elderly [8]. Thereby, effective life-saving treatments, e.g., lipid- and blood pressure lowering drugs, were applied more often and prescribed to older patients than before the reform [8, 44]. This more active medical treatment potentially explains the reversal in mortality trends from diabetes, stroke, pneumonia and symptoms and ill-defined conditions [8]. That the change in healthcare expenditures was not merely driven by higher prices of care was also confirmed by earlier analyses that ascribed about two-thirds of the increase to an extension of the volume of care [45].

The precise causal mechanisms how more spending translated into better survival in the Netherlands need to be understood in more detail. Recent studies revealed that particularly the survival of persons with more severe chronic conditions improved during the trend reversal of Dutch life expectancy, which indirectly confirms a possible impact of improved healthcare [46, 47]. However, so far the decline in mortality could not be linked to healthcare utilization at the individual level [46].

Although the impact of smoking did not explain the trend reversal in Dutch mortality it did affect trends in life expectancy. During the study period, the lower gains in life expectancy for Dutch women, as compared to Dutch men, were to a large extent caused by smoking; this is in line with the general theory of the smoking transition through which women progress with a delay of several decades as compared to men [48]. It has been reported that, after the exclusion of smoking-related causes of death, the stagnation in mortality decline occurred at the same time for men and women [7]. Our study results show that also the resumption of Dutch mortality decline after 2001 was similar for men and women, when the differential impact of smoking was accounted for. This concordance of sex-specific life expectancy trends before and after the Dutch trend reversal calls for a more general explanation that affected all Dutch inhabitants in the same way; we believe we have identified this in the form of financing of healthcare. No other plausible alternative explanation, that could have affected men and women in the same way, is available.

## Conclusions

The findings of this study highlight the growing importance of policy decisions regarding the allocation of healthcare resources. The case of the Netherlands demonstrates that changes in healthcare expenditures considerably affect trends in life expectancy.

## Additional file

Additional file 1:
**Technical background.** (DOC 1155 kb)

## Abbreviations

LE: Life expectancy
HCE: Healthcare expenditures
OECD: Organization for economic cooperation and development
GDP: Gross-domestic product
WHO: World Health Organization
CI: Confidence intervals
