# Supplementary material for: Quantifying the contribution of changes in healthcare expenditures and smoking to the reversal of the trend in life expectancy in the Netherlands
Source: BMC Public Health. 2015 Oct 6;15:1024. doi: 10.1186/s12889-015-2357-2 (PMC4596560; doi:10.1186/s12889-015-2357-2)
Supplement: Additional file 1: — Technical background. (DOC 1155 kb) [file 12889_2015_2357_MOESM1_ESM.doc]

**Additional file 1**

**A1. Modeling the effect of healthcare expenditures on life expectancy**

To estimate the effect of changes in healthcare expenditures (HCE) on changes in life expectancy (LE) we have specified a health-production function.[1, 2] Therein, monetary investments in health care as input were formally related to changes in health as output.

The starting point of our analysis was how HCE affects LE within a country over time. This allowed to assess whether an annual change in one variable did in fact co-occur with an annual change in the other variable.

Three challenges required particular attention for modeling such a relation. First, the effect is of stochastic nature so that a larger number of observations is necessary to make sure that the co-movement of the variables is not due to random variation only. Simulations have shown that time series with more than 50 years of observations are necessary to achieve stable results.[3] Second, changes in HCE only partly affect changes in LE immediately. The impact of healthcare investments on mortality is likely to have a delayed impact since new policies (e.g. prevention programs) and innovations (new medical technologies) require some time to enfold their full effects.[4] Third, to a certain extent unobserved variables drive the changes in HCE and LE so that a discovered effect might be spurious. This could be the diffusion of new expensive medical innovations (increasing HCE and LE) or a generally a shift in the health status of the population towards less severe and less costly diseases (decreasing HCE and increasing LE).

To solve these three issues the use of panel data is inevitable.[4] Panel data contain information for a group of countries on identical variables available for the same time span. This considerably extends the total number of observations thus resulting in more robust estimates. The time dimension of panel data allows detecting dynamic effects occurring with several years of delay.[5] The cross-sectional dimension of panel data enables to filter out unobserved country-specific effects influencing the relation of interest.[6]

However, using panel data poses additional challenges as the observations over time and countries are not independent from each other. The variables HCE and LE both trend upwards over time so that there is a considerable risk of detecting a link between the two, where actually no link exists.[7] Further, in a highly intertwined globalized world the countries are affected by common processes dependent on the cultural, geographical and economic proximity and similarity.[8] Finally, health system differentials among the countries result in a different ability to transform investments in health care into additional years of life.

To account for these caveats, we used a model specification that is able to deal with various sources of correlation in the data may distorting the estimation of the relation between HCE and LE. Moreover, our model is flexible enough to allow for a heterogeneous health production function among the countries while incorporating at the same time effects of common unobserved or omitted variables.[9] We compared our preferred specification with alternative models that inhibit less flexibility based on model fit and residual diagnostics. Furthermore, we performed extensive sensitivity analysis of our preferred model to ensure the robustness of our estimates.

**A2. Description of the data**

To estimate the health production function, we use as input healthcare expenditures expressed as proportion of gross domestic product (GDP) (and alternatively healthcare expenditures in US$ Purchasing Power Parity at 2005 prices) from the OECD Health Data 2014 and as output life expectancy at birth obtained from the Human Mortality Database.[10, 11] Information on per capita GDP at 2005 prices (excluding costs for health care), also from the OECD database, were used as confounder. Overall, our sample contained 19 countries spanning over 30 calendar years, as listed in table A1. We restricted our analysis to the period 1980-2009 because for this period complete information was available for almost any country (except France and Italy), while this time span still provides enough observations to detect dynamic effects. The country-specific means of life expectancy in this time span range from about 76 to 80 years (72 to 77 years in males and 79 to 83 years in females). The countries spend on average 6.9% to 10.2% of their total GDP on health care. Excluding these costs on health care, the time average of the GDP ranges from 16,010 US$ in Portugal to 34,861 US$ in Norway.

**Table A1-1. Mean values (arithmetic mean) over time between 1980 and 2009 for life expectancy, healthcare expenditures (per capita and as proportion of GDP) and GDP (excluding health care costs) in 19 OECD countries**

|  |  |  | Human Mortality Database | | | | OECD health data 2014 | | | |
| --- | --- | --- | --- | --- | --- | --- | --- | --- | --- | --- |
|  |  | availability | | LE  (total) | LE (males) | LE (females) | availability | HCE (per capita) | HCE (%GDP) | GDP |
| 1 | Australia | 1980-2009 | | 78.2 | 75.4 | 81.1 | 1980-2009 | 2144 | 7.4% | 26029 |
| 2 | Austria | 1980-2009 | | 76.7 | 73.4 | 79.7 | 1980-2009 | 2521 | 8.8% | 25281 |
| 3 | Belgium | 1980-2009 | | 76.7 | 73.5 | 79.9 | 1980-2009 | 2205 | 8.0% | 24665 |
| 4 | Canada | 1980-2009 | | 78.2 | 75.3 | 81.1 | 1980-2009 | 2651 | 9.0% | 26386 |
| 5 | Denmark | 1980-2009 | | 76.0 | 73.4 | 78.6 | 1980-2009 | 2508 | 8.9% | 25388 |
| 6 | Finland | 1980-2009 | | 76.6 | 72.7 | 80.3 | 1980-2009 | 1878 | 7.7% | 22400 |
| 7 | France | 1980-2009 | | 77.8 | 73.9 | 81.6 | 1990-2009 | 2804 | 10.2% | 24555 |
| 8 | Ireland | 1980-2009 | | 75.9 | 73.3 | 78.7 | 1980-2009 | 1770 | 7.1% | 22645 |
| 9 | Iceland | 1980-2009 | | 79.0 | 76.7 | 81.3 | 1980-2009 | 2359 | 8.4% | 27603 |
| 10 | Italy | 1980-2009 | | 78.1 | 74.9 | 81.2 | 1988-2009 | 2097 | 7.9% | 24179 |
| 11 | Japan | 1980-2009 | | 80.0 | 76.7 | 83.0 | 1980-2009 | 1883 | 7.1% | 24367 |
| 12 | The Netherlands | 1980-2009 | | 77.7 | 74.9 | 80.5 | 1980-2009 | 2550 | 8.6% | 26486 |
| 13 | New Zealand | 1980-2008 | | 76.7 | 74.0 | 79.4 | 1980-2009 | 1525 | 7.1% | 19465 |
| 14 | Norway | 1980-2009 | | 77.8 | 74.9 | 80.7 | 1980-2009 | 2950 | 8.1% | 34861 |
| 15 | Portugal | 1980-2009 | | 75.6 | 72.1 | 79.1 | 1980-2009 | 1373 | 7.5% | 16010 |
| 16 | Spain | 1980-2009 | | 78.3 | 74.8 | 81.6 | 1980-2009 | 1555 | 7.0% | 19987 |
| 17 | Sweden | 1980-2009 | | 78.6 | 76.0 | 81.2 | 1980-2009 | 2278 | 8.6% | 24030 |
| 18 | Switzerland | 1980-2009 | | 78.8 | 75.7 | 81.7 | 1980-2009 | 3132 | 9.2% | 30428 |
| 19 | United Kingdom | 1980-2009 | | 76.7 | 74.1 | 79.2 | 1980-2009 | 1812 | 6.9% | 23672 |
|  |  | min | | 75.6 | 72.1 | 78.6 | min | 1373 | 6.9% | 16010 |
|  |  | max | | 80.0 | 76.7 | 83.0 | max | 3132 | 10.2% | 34861 |
|  |  | span | | 4.4 | 4.6 | 4.4 | span | 1759 | 3.3% | 18851 |

A distinctive feature of healthcare expenditures is the high degree of correlation with GDP[12] and LE that is close to 1 (table A2). Put differently, as a country gets richer it tends to spend more on health care and at the same time people live longer. Due to the high degree of correlation it is hard to disentangle the effect of GDP and HCE on LE and at the same time such multicollinearity potentially inflates the variance in our regression model. For that purpose we used in our regression HCE expressed as proportion of GDP. For this indicator the correlation with GDP (r=0.66) and LE (r=0.84) is less strong than for HCE expressed in US$ (table A2).

**Table A1-2**. Bivariate correlations between the output and input variables in the health production function with country fixed effects, variables in natural logarithm

|  | LE | GDP | HCE US$ | HCE %GDP |
| --- | --- | --- | --- | --- |
| LE |  | 0.88 | 0.95 | 0.84 |
| GDP |  |  | 0.93 | 0.66 |
| HCE US$ |  |  |  | 0.89 |

**A3. Time series properties**

One of the reasons for the high degree of correlation between the variables we aim to put in the health production function is that they all strongly trend upward over time, probably because each variable is also a proxy for general societal progress. In technical terms variables that do not reverse to their mean are non-stationary because they contain a unit root. In such a case, the estimates of classical OLS approaches are subject to the risk of being spurious.[13] To detect the existence of a possible unit-root process in our variables, we tested for non-stationarity in our panel. A flexible test is the CIPS allowing cross-sectional heterogeneity and unbalanced data in the sample.[14] Results of this test are shown in table A3, where up to 4 lags were included to account for serial correlation. The test suggests the existence of a unit root with and without assuming a trend in the series. In particular the series are outcomes of a process integrated of order one, since the null hypothesis of all countries containing a unit-root was not rejected in levels but rejected in differences for most specifications. We performed also a simpler panel unit root that does not account for cross-sectional dependence in the panel as suggested by Maddala & Wu (1999) with virtually the same results.[15]

**Table A3-1.** Panel unit-root test for output and input variables in the health production function

|  | LE |  | GDP |  | HCE US$ |  | HCE %GDP |  |
| --- | --- | --- | --- | --- | --- | --- | --- | --- |
| without trend: | ztbar | p | ztbar | p | ztbar | p | ztbar | p |
| lags: 0 | **-4.4** | 0.00 | 1.8 | 0.97 | -1.1 | 0.13 | 0.8 | 0.79 |
| 1 | -0.7 | 0.24 | -0.5 | 0.32 | -1.2 | 0.11 | 0.3 | 0.62 |
| 2 | -1.2 | 0.12 | 1.6 | 0.94 | -0.2 | 0.41 | 1.5 | 0.93 |
| 3 | -1.0 | 0.16 | 1.0 | 0.84 | 0.1 | 0.55 | 2.0 | 0.98 |
| with trend: |  |  |  |  |  |  |  |  |
| lags: 0 | **-3.6** | 0.00 | 3.9 | 1.00 | 1.0 | 0.83 | 2.7 | 1.00 |
| 1 | 0.5 | 0.68 | 1.9 | 0.97 | 1.3 | 0.90 | 2.5 | 0.99 |
| 2 | 0.4 | 0.65 | 4.1 | 1.00 | 3.0 | 1.00 | 4.1 | 1.00 |
| 3 | -0.4 | 0.36 | 3.6 | 1.00 | 3.9 | 1.00 | 4.7 | 1.00 |
| in differences: |  |  |  |  |  |  |  |  |
| lags: 0 | **-18.3** | 0.00 | **-8.0** | 0.00 | **-11.4** | 0.00 | **-11.1** | 0.00 |
| 1 | **-9.4** | 0.00 | **-6.2** | 0.00 | **-6.8** | 0.00 | **-6.5** | 0.00 |
| 2 | **-4.0** | 0.00 | -1.5 | 0.07 | **-3.7** | 0.00 | **-3.0** | 0.00 |
| 3 | **-2.6** | 0.01 | -0.6 | 0.26 | -1.2 | 0.11 | -0.8 | 0.21 |

**Note: bold values indicate significant values at p<0.05, thus rejecting the null of nonstationarity**

**A4. Model building**

*Estimation with the variables in levels (LEVELS)*

Following Baltagi et al 2011 and Skinner and Staiger 2009, we define a Cobb-Douglas production function, where the output is life expectancy at birth (LE) while healthcare expenditures (HCE) proxy the bundle of the inputs capital and labor.[8, 16]


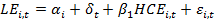
 (1a)

All variables in (1a) are in logs to estimate the elasticity of input and output, but also to account fo r a decreasing return of marginal investments and to guard the model against the influence of outliers. The subscripts *i* and *t* denote country and time, *α* represents stable differences in medical technology between countries and *δ* the progress of medical technology over time common in all countries. Finally, *β_1_* represents the percentage change in LE with respect to a percentage change in HCE common in all countries. This specification is denoted as LEVELS since it assumes that a higher level of HCE corresponds to a higher level of LE.

*Estimation with the variables in first differences (FD)*

Since we have demonstrated in A2 that the variables in our regression are non-stationary in levels but stationary in first differences, we should favor the estimation of a relation between LE and HCE with the variables in first differences, as shown in (1b). This enables to avoid the risk of a spurious correlation in regressions with non-stationary variables.[13]


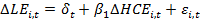
 (1b)

*Dynamic pooled two-way fixed effect model (2FE)*

However, estimating the relation between HCE and LE in first differences would remove any long-run relationship between LE and HCE.[17] Since theoretical reasoning above suggested that investments in health care partially also affect mortality with a certain delay, removing long-run effects of HCE on LE would not adequately catch the dynamic impact of changes in health care spending. Therefore, we have decided for an error-correction model, where the long-run relationship of the variables in levels is added to the right-hand side of equation (1b) resulting in equation (2). This is able to measure a dynamic response of LE to changes in HCE divided into two parts.[18] First, changes in HCE could directly initiate changes in LE during the same period. Second, an increase in HCE may results in a long-term response of LE until the equilibrium relationship between HCE and LE is restored. In difference to other dynamic models where an finite number of lags has to be specified a priori, the error-correction model allows for a flexible response of LE to a change in HCE without a prior specification of the particular lag time merely assuming that the effect declines geometrically over time.[5] Further, since the model disentangles a short-run and long-term relation between the variables in the health production function it is - unlike the classical linear static regression - suited for both stationary and non-stationary data.[5, 18]


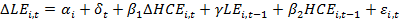
 (2)

In the error-correction specification as expressed in (2), *β_1_* tests for the immediate response of LE to a change in HCE during the same year, thus the short-run effect. The second coefficient of HCE *β_2_* expresses the combined effect on LE during the same year and the next year, while the combination of *β_2_* and *γ* represent the long-run effect, computed as *β_2_*/*γ.* If *γ* and *β_2_* are significantly greater than zero, a long-run relation between the variables exists.[18] Otherwise the model reduces to short-run relation between the changes in LE and HCE only, given that *β_1_* is significant and greater than zero, which is equivalent to the model estimated with the variables in first differences. Equation (2) contains fixed effects for countries and calendar years and restricts the coefficients of HCE to be the same for all countries. For this reason the model is termed the pooled two-way fixed effects model (2FE).

*Dynamic mean-group estimator (MG)*

Although the 2FE in (2) allows for a dynamic relationship between HCE and LE it is still a quite restrictive specification, given that it assumes common health technology among all countries, i.e. that a similar investment in health care results in a similar increase in life expectancy. Moreover, the time series of each country were treated as independent from each other. A more realistic but also more complex specification is to assume that each country has not only its own intercept as in (2) but also its own time trend and effect of HCE (and HDP) on LE. For this purpose the relation between the variables and fixed effects is estimated in a first step in each country separately as depicted in (3). In a second step, the coefficients are averaged (3a) and tested for the null hypothesis that the average equals 0, denoted as mean-group estimator (MG).[19]


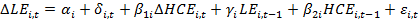
 (3)


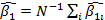
 (4)

*Dynamic correlated mean-group estimator (CMG)*

The mean-group estimator relaxes the assumption of a homogeneous health technology, but still assumes that the health production among the countries operates independently from each other. An intermediate solution between pooling all countries and estimating the regressions completely separately is to introduce common factors representing global shocks and local spillover effects.[20]


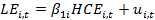
 (5)


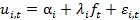
 (6)


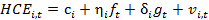
 (7)

Compared to (1) the model specifications in (5) and (6) and (7) additionally contain the unobserved common factor *f* correlated with the error term in (5) but also with the explanatory variable HCE in (7), which is additionally driven by a second factor *g*. The responses to these factors are country-specific, denoted as *λ_i_*, *η_i_* and
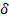
*_i_*.[20] This approach models unobservable or omitted variables that confound the relation between LE and HCE that are common in all countries but have a different impact in each country such as economic recessions or new medical innovations. This framework is in line with earlier findings that the development of life expectancy and of healthcare expenditures is mainly driven by a shared progress in technology.[16, 21, 22] Controlling for the influence of this should reveal the impact of HCE on LE.

To incorporate the effect of unobserved common factors in the dynamic regression model (3) we follow Pesaran 2006 who demonstrated that the inclusion of cross-sectional averages of all variables in the model is a sufficient proxy for the factors.[6] In the basic specification model (3) is augmented with the cross-sectional averages of all variables, presented in (8). This model is specified as correlated mean-group estimator (CMG)


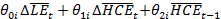
 (8)

*Dynamic correlated mean-group estimator with distance weights (CMGD)*

The standard CMG as explained above assumes that for any country all the other countries in the sample are of equal importance to proxy the unobserved factors influencing LE and HCE. As convincingly shown by Baltagi et al 2012, a more plausible assumption is that closer countries are more relevant for developments in a particular country than more distant countries.[8] This enables to incorporate spatial dependence in the model, like common health policies, regional weather extremes or shared cultural, economic and genetic characteristics among a group of countries. An effective way to incorporate such a proximity in the estimation of the production function is to weight the observations in the countries before constructing the cross-sectional means in (8) by the inverse of the distance between a pair of countries.[8] The construction of the weights is described in detail elsewhere.[23] The distance-weighted version of the CMG will be denoted as CMGD.

*Confounders*

As mentioned in A2 the effects of HCE and GDP are hard to disentangle. For this reason we add GDP as central confounder to all models since otherwise the effect of changes in HCE would to a certain extent measure changes in GDP.[12, 24] The empirical implementation of an additional explanatory variable in the models described above is similar to the inclusion of HCE. We do not add further confounders to our models for two reasons. First, the influence of omitted and unobservable varaibles is indirectly included in our models either as fixed effects or as common factors. Second, the data quality and availability of other confounders is much worse than for the variables HCE and GDP so that inclusion of them would potentially do more harm than good and reduce the sample size drastically. Nevertheless, in the sensitivity analysis of our preferred model, we will test the model robustness to the inclusion of further variables.

A central assumption of all models introduced in this section is that remaining residuals are white noise. For this purpose we performed test for remaining temporal and spatial correlation by performing the Pesaran CIPS panel-unit root test for the presence of non-stationary and the Pesaran CD-test for cross-sectional independence of the residuals.[14, 25] In line with a number of recent contributions in the field of panel econometrics, our goal is to discover and incorporate the sources of violation of the assumptions of OLS regressions rather than correcting away these violations as it was a common practice in the past.[4, 23, 26] Rather than relying on a single model, we test all specifications explained above and favor the one that is theoretically most plausible but at the same time provides a good fit to the data and well-behaved residuals.

**A5. Results**

The results of the six model specifications are shown in table A4(a). In models 1 to 3 the effect of HCE on LE is estimated in the pooled dataset, while in models 4 to 6 the estimators of the country-specific regressions were averaged. The static models 1 and 2 estimate either the immediate or long-run effect, while the dynamic models 3 to 6 contain both aspects and provide the speed until the full impact is visible guided by the coefficient of error correction. The proportion of the total effect that occurs in the first two periods (t=0 and t=1) is shown below the long-run effect. The model fit is expressed as root mean squared error (RMSE) and the results of the tests for stationary and cross-sectional independence of the residuals are displayed at the bottom of the table. Thereby, we report whether the CIPS test with up to 3 lags with and without trends rejects the null of nonstationary of the residuals and display the absolute mean cross-sectional correlation of the residuals in line with the CD test statistic for cross-sectional independence.

The comparison of the models offers interesting insights into the influence of different specifications. We find evidence for a long-run relationship already in the least complex model with variables in levels (column 1 in table A4(a)). An increase of one percent of HCE corresponds to a change in LE by 0.022 percent. The separate regression of the annual changes of HCE and LE in model 2 does not indicate an immediate relationship. The residual diagnostics of the two static models reveal remaining correlation in the temporal and spatial dimension signalling a possible misspecification. Moving to the pooled dynamic model confirms the presence of a long-run effect (elasticity of 0.028) and the absence of an immediate effect. The error correction is estimated to be 0.225, which means that the initial short-run effect of HCE (0.006) diminished by 22.5% every year so that at the end of the second period only a fifths of the total effect occurred. Although the pooled 2FE model achieves a better model fit than the two static models, the residual diagnostics are still unfavourable.

**Table A5-1 Short-run and long-run effect of a change in healthcare expenditures as % of GDP on life expectancy at birth (standard errors in parentheses) in six different specifications,**

|  | (1) | (2) | (3) | (4) | (5) | (6) |
| --- | --- | --- | --- | --- | --- | --- |
| SPECIFICATION | LEVELS  pooled static | FD  pooled static | 2FE  pooled dynamic | MG  averaged dynamic | CMG  averaged dynamic | CMGD  averaged dynamic |
|  |  |  |  |  |  |  |
| Immediate effect (t=0) |  | -0.004 | 0.003 | 0.005 | 0.019** | 0.010 |
|  |  | (0.005) | (0.004) | (0.006) | (0.007) | (0.006) |
| Error correction |  |  | -0.225*** | -0.802*** | -0.850*** | -0.539*** |
|  |  |  | (0.027) | (0.073) | (0.078) | (0.089) |
| Short-run effect(t=0 & t=1) |  |  | 0.006** | 0.014 | 0.024** | 0.020** |
|  |  |  | (0.002) | (0.008) | (0.008) | (0.008) |
| Long-run effect | 0.022*** |  | 0.028*** | 0.017 | 0.028** | 0.036** |
|  |  |  | (0.008) | (0.010) | (0.009) | (0.012) |
| proportion of the long-run effect at the end of t=1: |  |  | 21% | 82% | 82% | 56% |
|  |  |  |  |  |  |  |
| Observations | 513 | 513 | 513 | 513 | 513 | 513 |
| RMSE | 0.0046 | 0.0032 | 0.0028 | 0.0023 | 0.0016 | 0.0018 |
|  |  |  |  |  |  |  |
| Residuals:  Stationary  Mean |*p*|  CD statistic (*p*) | NO  0.424***  4.41 | NO  0.308***  17.12 | NO  0.226*  -2.42 | YES  0.221***  10.69 | YES  0.212**  -3.00 | YES  0.203  0.15 |

Standard errors in parentheses

*** p<0.001, ** p<0.01, * p<0.05

Note: All models contain life expectancy at birth, per capita healthcare expenditures and per capita GDP (excluding costs for health care). LEVELS and FD estimate pooled static regressions with time and country dummies, 2FE denotes the pooled two-way fixed effects model including country and time dummies, while MG/CMG/CMGD denote each the mean group estimator with no weights/equal weights/distance weights where the model is fit separately fit to any country and then averaged. To preserve an equal number of observations and the full sample size of 19 countries we have not added further lags of the cross-sectional averages in model 6+7.

**Table A5-2 Country-specific long-run effects of a change in healthcare expenditures as % of GDP on life expectancy at birth underlying model (6) in table A4(a)**

| Country | Long-run effect |
| --- | --- |
| 1. Belgium | 0.1194 |
| 2. Ireland | 0.0958 |
| 3. Japan | 0.0880 |
| 4. Denmark | 0.0684 |
| 5. Australia | 0.0655 |
| *6. Netherlands* | *0.0592* |
| 7. New Zealand | 0.0554 |
| 8. Iceland | 0.0482 |
| 9. France | 0.0426 |
| **Model (6) CMGD** | **0.0360** |
| 10. Canada | 0.0179 |
| 11. Austria | 0.0146 |
| 12. Italy | 0.0004 |
| 13. Portugal | -0.0030 |
| 14. Switzerland | -0.0075 |
| 15. United Kingdom | -0.0087 |
| 16. Spain | -0.0092 |
| 17. Sweden | -0.0105 |
| 18. Norway | -0.0336 |
| 19. Finland | -0.0422 |

Allowing for heterogeneous health technology by fitting separate regressions for any country (model 4) solves the problem of remaining non-stationary residuals, reduces the spatial correlation to 0.221 and further improves the model fit to a RMSE of 0.00023. The averaged coefficients of the effects of HCE are not significant anymore. By contrast, as soon as we introduce common factors to the separate regressions (model 5) both the immediate and the long-run effect of HCE becomes highly significant and the model fit improved remarkably to an error of 0.0016. However, the residuals still exhibit spatial dependence. Finally, the model where the relevance of the common factors depend on the geographical distance of a country to the other countries of the sample (model 6) is the only one with favorable residual diagnostics – both for temporal and spatial correlation. This specification results in an effect of HCE of 0.036 in the long-run. About half of this total effect (56%) occurs already in the first two periods at t=0 and t=1 (0.02). The strength of the effect diminishes every year by about 46%, computed as 1-0.539.

Based on these results, we decided to use the coefficients of the more flexible and well-specified CMGD models for computing the contribution of the change in healthcare expenditures on the change in life expectancy at birth in the main analysis of our paper. Given the complexity of the dynamic model, we provide a visualization of its mechanism in figure A1 demonstrating how a single 10 percent change in healthcare expenditures at t=0 increases life expectancy in the subsequent years. During the first year life expectancy at birth (we assume a level of 80 years at t=0) grows immediately by 0.1% or 0.08 years, while this is slightly larger in the next year. Subsequently, the effect size weakens fast and after about 5 years almost the full effect of 0.26% has been reached corresponding to an increase of life expectancy of 0.3 years.

**Figure A5-1 Annual and cumulative change in life expectancy at birth given a 10% change in healthcare expenditures in t=0, based on the dynamic model 6 in table A4**


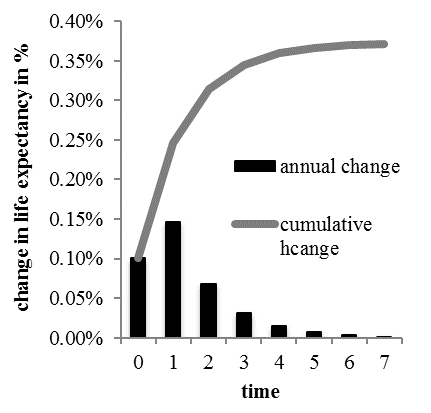

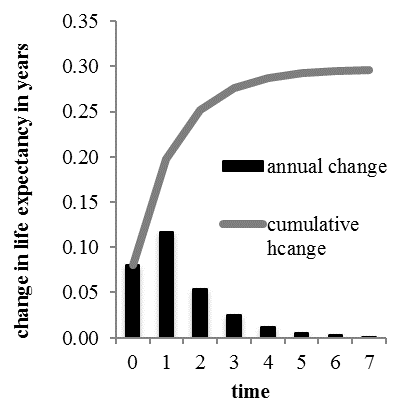


**A6. Sensitivity analysis**

We have performed extensive sensitivity analyses to assess the robustness of our preferred model specification (CMGD, model 6 in table A4). The results of this model are again shown in column 1 of table A5 compared with the results of eight alternative specifications (model 7-14). In model 7, we have added two additional lags of the cross-sectional averages in equation (8) as generally suggested for smaller samples to ensure the validity of the estimates, which is at the cost of reducing the number of observations from 513 to 441.[27] To check the influence of the selected time span 1980-2009, we have also fitted our preferred model specification to the full time span available in the 2014 OECD health database, shown in model 8. In model 9 we added the variables per capita alcohol consumption and the age-standardized lung cancer death rate, which has been demonstrated to be an excellent proxy for the cumulative effect of smoking.[28] Model 10 estimates the model without taking the natural logarithm of the variables. Further, in model 11 we have excluded the Netherlands to see whether the effect of HCE is also visible without this deviating country. In addition, we have checked the sensitivity of the results with respect to the exclusion of any other country in the sample, shown in figure A2. In model 12 and 13 in table A5 we have used life expectancy of males and females only. In model 13 we have used health care expenditures measures in per capita US$ at 2005 prices and constant purchasing power parities.

In sum, all alternative specifications confirm the existence of a short-run and long-run relationship between LE and HCE in the CMGD specification, while an immediate effect was only visible in 4 of the 8 alternative specifications. The total effect of HCE varied between 0.15 and 0.53 so that our preferred estimate of 0.36 ranges in the middle of these extremes. The proportion of the total effect after two periods ranged between 28% and 91% compared to 56% in our preferred model specification.

**Table A6-1 Short-run and long-run effect of a change in healthcare expenditures as % of GDP on life expectancy at birth in alternative specifications**

|  | (6) | (7) | (8) | (9) | (10) | (11) | (12) | (13) | (14) |
| --- | --- | --- | --- | --- | --- | --- | --- | --- | --- |
| ALTERNATIVE SPECIFICATION | CMGD  averaged dynamic | With 2 additional CS lags | Maximum time span 1960-2013 | + alcohol consumption, lung cancer | Variables not in log | NLD excluded | Male life expectancy | Female life expectancy | HCE in per capita US$ |
| Immediate effect; t=0 | 0.010 | 0.042* | 0.009* | 0.026 | 0.135** | 0.016* | 0.013 | 0.001 | 0.012 |
|  | (0.006) | (0.020) | (0.004) | (0.017) | (0.047) | (0.006) | (0.008) | (0.006) | (0.006) |
| Error correction | -0.553*** | -0.957*** | -0.288*** | -0.717*** | -0.645*** | -0.565*** | -0.504*** | -0.664*** | -0.601*** |
|  | (0.097) | (0.168) | (0.067) | (0.120) | (0.086) | (0.090) | (0.083) | (0.093) | (0.090) |
| Short-run effect; t=0 & t=1 | 0.020** | 0.053* | 0.015** | 0.032* | 0.187* | 0.020* | 0.024* | 0.019* | 0.016* |
|  | (0.008) | (0.025) | (0.006) | (0.014) | (0.061) | (0.008) | (0.009) | (0.007) | (0.007) |
|  |  |  |  |  |  |  |  |  |  |
| Long-run effect | 0.036** | 0.058* | 0.053** | 0.045** | 0.029** | 0.035** | 0.047** | 0.028* | 0.026** |
|  | (0.012) | (0026) | (0.018) | (0.019) | (0.091) | (0.0123) | (0.015) | (0.010) | (0.011) |
| proportion of the long-run effect at the end of t=1: | 56% | 91% | 28% | 71% | 64% | 57% | 51% | 68% | 62% |
|  |  |  |  |  |  |  |  |  |  |
| Observations | 513 | 441 | 702 | 463 | 513 | 485 | 513 | 513 | 513 |
| RMSE | 0.0018 | 0.0010 | 0.0024 | 0.0011 | 0.14 | 0.0018 | 0.0022 | 0.0029 | 0.0018 |
|  |  |  |  |  |  |  |  |  |  |

Standard errors in parentheses

*** p<0.001, ** p<0.01, * p<0.05

**Figure A6-1 Robustness of the estimated long-run relationship between healthcare expenditures as % of GDP and life expectancy at birth from model 6 in table A4 with respect to the exclusion of a country at a time from the sample**

**A7. Calculation of the impact of smoking and healthcare expenditures for other countries with reversals of trends in life expectancy**

**Table A7-1. Decennial change in life expectancy (LE) at birth and contribution of smoking and healthcare expenditures (HCE) in Denmark and Ireland, 1990-1999 and 2000-2009**

|  | **period** | **observed change in LE** | **diff 1990-99 and 2000-09** | **change in LE due to changes in smoking** | **diff 1990-99 and 2000-09** | **change in LE due to changes in HCE** | **diff 1990-99 and 2000-09** |
| --- | --- | --- | --- | --- | --- | --- | --- |
|  |  |  |  |  |  |  |  |
|  |  |  |  |  |  |  |  |
| **females** |  | Denmark | | | | | |
|  | **1990-99** | 1.2 | **0.7** | -0.4 |  | 0.0 |  |
|  | **2000-09** | 1.9 |  | -0.2 | **0.2** | 0.5 | **0.5** |
|  |  | Ireland | | | | | |
|  | **1990-99** | 1.2 | **1.8** | 0.2 |  | 0.1 |  |
|  | **2000-09** | 3.0 |  | 0.0 | **-0.2** | 1.0 | **0.9** |
| **males** |  | Denmark | | | | | |
|  | **1990-99** | 2.2 | **0.2** | 0.5 |  | 0.0 |  |
|  | **2000-09** | 2.4 |  | 0.4 | **-0.1** | 0.5 | **0.5** |
|  |  | Ireland | | | | | |
|  | **1990-99** | 1.3 | **2.0** | 0.7 |  | 0.1 |  |
|  | **2000-09** | 3.3 |  | 0.4 | **-0.4** | 1.0 | **0.9** |

**A8. Sensitivity analysis using a different approach to estimate the impact of smoking on mortality (as suggested by Preston et al. 2010) and a different indicator for healthcare expenditures (expressed in per capita US$)**

**Table A8-1. Decennial change in life expectancy (LE) at birth and contribution of smoking (using the method of Preston et al. 2010) and healthcare expenditures (expressed in per capita US$) in the Netherlands and the mean of 18 other OECD countries, 1990-1999 and 2000-2009**

|  | **period** | **observed change in LE** | **change 1990-99 and 2000-09** | **change in LE due to changes in smoking** | **change 1990-99 and 2000-09** | **change in LE due to changes in HCE** |  | **change 1990-99 and 2000-09** |  |
| --- | --- | --- | --- | --- | --- | --- | --- | --- | --- |
|  |  |  |  |  |  |  |  |  |  |
|  |  |  |  |  |  |  | **95% CI** |  | **95% CI** |
| **females** |  | Netherlands | | | | | | | |
|  | **1990-99** | 0.3 | **1.8** | -0.4 |  | 0.4 | (0.02 to 0.72) |  |  |
|  | **2000-09** | 2.1 |  | -0.4 | **0.0** | 1.0 | (0.02 to 1.84) | **0.6** | (0.00 to 1.14) |
|  |  | mean of the other countries | | | | | | | |
|  | **1990-99** | 1.7 |  | -0.2 |  | 0.5 | (0.03 to 0.97) |  |  |
|  | **2000-09** | 1.9 | **0.2** | -0.2 | **0.1** | 0.6 | (0.00 to 1.20) | **0.1** | (-0.04 to 0.29) |
| **males** |  | Netherlands | | | | | | | |
|  | **1990-99** | 1.5 | **1.5** | 0.5 |  | 0.4 | (0.02 to 0.67) |  |  |
|  | **2000-09** | 3.0 |  | 0.7 | **0.2** | 0.9 | (0.02 to 1.84) | **0.5** | (0.00 to 1.05) |
|  |  | mean of the other countries | | | | | | | |
|  | **1990-99** | 2.2 |  | 0.1 |  | 0.5 | (0.02 to 0.89) |  |  |
|  | **2000-09** | 2.5 | **0.3** | 0.2 | **0.1** | 0.6 | (0.00 ot 1.10) | **0.1** | (0.00 to 0.26) |

**A9. Sensitivity analysis using a different time span to compare changes in the impact of smoking and healthcare on mortality (1994-2001 vs. 2002-2009)**

Table A9-1. Change in life expectancy (LE) at birth and contribution of smoking and healthcare expenditures (HCE) in the Netherlands and the mean of 18 other OECD countries, 1994-2001 and 2002-2009

|  | **Period** | **Observed change in LE** | **Difference between 1994-2001 and 2002-2009** | **Change in LE due to changes in smoking** | **Difference between 1994-2001 and 2002-2009** | **Change in LE due to changes in HCE** |  | **Difference between 1994-2001 and 2002-2009** |  |
| --- | --- | --- | --- | --- | --- | --- | --- | --- | --- |
|  |  |  |  |  |  |  |  |  |  |
|  |  |  |  |  |  |  | **95% CI** |  | **95% CI** |
| **females** |  | The Netherlands | | | | | | | |
|  | **1994-01** | 0.4 | **1.6** | -0.4 |  | -0.1 | (-0.15 to -0.01) |  |  |
|  | **2002-09** | 2.0 |  | -0.4 | **0.0** | 0.8 | (0.27 to 1.39) | **0.9** | (0.29 to 1.52) |
|  |  | Mean of the other countries | | | | | | | |
|  | **1994-01** | 1.4 |  | -0.1 |  | 0.2 | (0.05 to 0.29) |  |  |
|  | **2002-09** | 1.5 | **0.1** | -0.1 | **-0.1** | 0.3 | (0.10 to 0.50) | **0.2** | (0.05 to 0.27) |
| **males** |  | The Netherlands | | | | | | | |
|  | **1994-01** | 1.2 | **1.3** | 0.6 |  | -0.1 | (-0.14 to -0.01) |  |  |
|  | **2002-09** | 2.6 |  | 0.4 | **-0.3** | 0.8 | (0.25 to 1.29) | **0.8** | (0.27 to 1.41) |
|  |  | Mean of the other countries | | | | | | | |
|  | **1994-01** | 1.8 |  | 0.3 |  | 0.2 | (0.05 to 0.27) |  |  |
|  | **2002-09** | 1.9 | **0.1** | 0.1 | **-0.1** | 0.3 | (0.10 to 0.50) | **0.1** | (0.05 to 0.25) |

References

1. Lichtenberg FR: **Sources of US longevity increase, 1960-2001**. *The quarterly review of economics and finance* 2004, **44**(3):369-389.

2. Or Z: **Determinants of health outcomes in industrialised countries: a pooled, cross-country, time-series analysis**. *OECD Econ Stud* 2000:53-78.

3. Pedroni P: **Social capital, barriers to production and capital shares: implications for the importance of parameter heterogeneity from a nonstationary panel approach**. *J Appl Econometrics* 2007, **22**(2):429-451.

4. Reibling N: **The international performance of healthcare systems in population health: Capabilities of pooled cross-sectional time series methods**. *Health Policy* 2013.

5. Beck N, Katz JN: **Modeling dynamics in time-series-cross-section political economy data**. *Annual Review of Political Science* 2011, **14**:331-352.

6. Pesaran MH: **Estimation and inference in large heterogeneous panels with multifactor error structure**. *Econometrica* 2006, **74**:967-1012.

7. Akkoyunlu S, Lichtenberg FR, Siliverstovs B, Zweifel P: **Spurious correlation in estimation of the health production function: A note**. *Economics Bulletin* 2010, **30**(3):2505-2514.

8. Baltagi BH, Moscone F, Tosetti E: **Medical technology and the production of health care**. *Empirical Econ* 2012, **42**(2):395-411.

9. Eberhardt M, Teal F: **The Magnitude of the Task Ahead: Productivity Analysis With Heterogeneous Technology**. 2014.

10. OECD: **OECD Health Statistics 2014**. 2014.

11. Human Mortality Database: **University of California, Berkeley (USA), and Max Planck Institute for Demographic Research (Germany)**. In*.*; 2014.

12. Gravelle H, Jacobs R, Jones AM, Street A: **Comparing the efficiency of national health systems: a sensitivity analysis of the WHO approach**. *Applied Health Economics and Health Policy* 2003, **2**(3):141-148.

13. Granger CW, Newbold P: **Spurious regressions in econometrics**. *J Econometrics* 1974, **2**(2):111-120.

14. Pesaran MH: **A simple panel unit root test in the presence of cross‐section dependence**. *J Appl Econometrics* 2007, **22**(2):265-312.

15. Maddala GS, Wu S: **A comparative study of unit root tests with panel data and a new simple test**. *Oxford Bull Econ Statist* 1999, **61**(S1):631-652.

16. Skinner J, Staiger D: **Technology diffusion and productivity growth in health care**. In*.*: National Bureau of Economic Research; 2009.

17. Herzer D, Strulik H: **Religiosity and income a panel cointegration and causality analysis**. In: *Discussion papers / Center for European Governance and Economic Development Research 168.* Göttingen: CeGE

Niedersächsische Staats- und Universitätsbibliothek; 2013.

18. Keele L, De Boef S: **Not just for cointegration: error correction models with stationary data**. *Department of Politics and International Relations, Nuffield College and Oxford University* 2004.

19. Pesaran MH, Smith R: **Estimating long-run relationships from dynamic heterogeneous panels**. *J Econometrics* 1995, **68**(1):79-113.

20. Eberhardt M, Presbitero A: **This Time They Are Different: Heterogeneity and Nonlinearity in the Relationship Between Debt and Growth**. In: *IMF Working Paper.* International Monetary Fund; 2013.

21. Oeppen J, Vaupel JW: **Broken limits to life expectancy**. *Science* 2002, **296**(5570):1029.

22. White KM: **Longevity advances in high-income countries, 1955-96**. *Population Devel Rev* 2002, **28**(1):59-76.

23. Eberhardt M, Teal F: **No Mangos in the Tundra: Spatial Heterogeneity in Agricultural Productivity Analysis**. *Oxford Bull Econ Statist* 2012.

24. Heijink R, Koolman X, Westert GP: **Spending more money, saving more lives? The relationship between avoidable mortality and healthcare spending in 14 countries**. *The European Journal of Health Economics* 2012.

25. Pesaran MH: **General Diagnostic Tests for Cross Section Dependence in Panels**. *IZA Discussion Paper* 2004, **No. 1240**.

26. Eberhardt M, Teal F: **Econometrics For Grumblers: A New Look At The Literature On Cross‐Country Growth Empirics**. *J Econ Surveys* 2011, **25**(1):109-155.

27. Chudik A, Pesaran MH: **Common Correlated Effects Estimation of Heterogeneous Panel Data Models with Weakly Exogeneous Regressors**. *Federal Reserve Bank of Dallas Globalization and Monetary Policy Institute Working Paper* 2013, **No. 146**.

28. Preston SH, Glei DA, Wilmoth JR: **A new method for estimating smoking-attributable mortality in high-income countries**. *Int J Epidemiol* 2010, **39**(2):430-438.
